# Oxidative Stress in Maternal and Offspring Kidney Disease and Hypertension: A Life-Course Perspective

**DOI:** 10.3390/antiox14040387

**Published:** 2025-03-26

**Authors:** Pei-Chen Lu, You-Lin Tain, Ying-Jui Lin, Chien-Ning Hsu

**Affiliations:** 1 Division of Pediatric Nephrology, Kaohsiung Chang Gung Memorial Hospital, Kaohsiung 833, Taiwan; latina@cgmh.org.tw (P.-C.L.); tainyl@cgmh.org.tw (Y.-L.T.); 2 Department of Pediatrics, Kaohsiung Municipal Ta-Tung Hospital, Kaohsiung 801, Taiwan; 3 College of Medicine, Chang Gung University, Taoyuan 333, Taiwan; 4 Division of Critical Care, Department of Pediatrics, Kaohsiung Chang Gung Memorial Hospital and Chang Gung University College of Medicine, Kaohsiung 833, Taiwan; rayray@adm.cgmh.org.tw; 5 Division of Cardiology, Department of Pediatrics, Kaohsiung Chang Gung Memorial Hospital and Chang Gung University College of Medicine, Kaohsiung 833, Taiwan; 6 Department of Early Childhood Care and Education, Cheng Shiu University, Kaohsiung 833, Taiwan; 7 Department of Respiratory Therapy, Kaohsiung Chang Gung Memorial Hospital and Chang Gung University College of Medicine, Kaohsiung 833, Taiwan; 8 Department of Pharmacy, Kaohsiung Chang Gung Memorial Hospital, Kaohsiung 833, Taiwan; 9 School of Pharmacy, Kaohsiung Medical University, Kaohsiung 807, Taiwan

**Keywords:** kidney disease, hypertension, oxidative stress, reactive oxygen species, antioxidant, nitric oxide, asymmetric dimethylarginine, Developmental Origins of Health and Disease (DOHaD), developmental programming

## Abstract

Kidney disease and hypertension are interconnected, prevalent conditions that affect both pregnant women and children. Oxidative stress occurs when reactive oxygen species or reactive nitrogen species exceed the capacity of antioxidant systems. It plays a critical role in kidney development, resulting in kidney programming and increased risks for kidney disease and hypertension across the life course. Animal models have significantly advanced our understanding of oxidative stress-related kidney programming, the molecular mechanisms involved, and early-life antioxidant interventions to prevent kidney disease. This review critically examines the influence of perinatal oxidative stress on kidney development, highlighting its long-term effects on kidney outcomes and susceptibility to hypertension. It also explores the potential of antioxidant-based interventions in preventing kidney disease and hypertension. Furthermore, the review addresses the existing gap between insights gained from animal models and their translation into clinical practices, emphasizing the challenges and opportunities for future research in this area.

## 1. Introduction

The fetal and infant stages of life are decisive for future health. Adverse environmental exposures during this period can have lasting detrimental effects on offspring throughout their lifetime [1,2]. Developmental programming occurs when such exposures alter the structure and function of fetal organ systems, increasing the risk of diseases in adulthood [3]. These insights led to the emergence of the research field known as the Developmental Origins of Health and Disease (DOHaD) [4]. Remarkably, the life course approach informs high-risk prevention strategies for adult diseases, beginning in the prenatal and early postnatal stages [5]. This approach enables a shift in therapeutic interventions from adulthood to fetal or infant life, a process known as reprogramming [6,7]. As a result, reprogramming has the potential to work as an innovative preventive strategy, reducing the global burden of many chronic diseases.

Oxidative stress has emerged as a key link between adverse early-life environmental factors and disorders related to DOHaD [8,9,10]. This imbalance, resulting from the overproduction of reactive oxygen and nitrogen species (ROS and RNS) that exceed the capacity of cellular antioxidant defenses [11], has been observed in both human and experimental studies involving suboptimal in utero conditions during fetal development [12,13]. Conversely, findings from animal models indicate that antioxidants could hold potential for the early-life prevention and therapeutic intervention of diseases related to DOHaD in adult offspring [8,9,10].

Chronic kidney disease (CKD) and hypertension are highly prevalent conditions, including among pregnant women [14]. Globally, CKD affects an estimated 6% of women of reproductive age and occurs in approximately 3% of pregnancies [15]. Hypertensive disorders complicate 5–10% of pregnancies, significantly impacting maternal and offspring outcomes [16]. CKD and hypertension are closely interconnected [17], with both conditions often originating in early life [18,19,20]. Women with CKD face an increased risk of adverse pregnancy outcomes [21], and hypertension is a major contributor to these risks.

Pregnancy heightens susceptibility to oxidative stress due to elevated ROS/RNS and systemic inflammation [22]. While enzymatic and non-enzymatic defense systems help counteract ROS/RNS, excessive levels can lead to adverse maternal and fetal outcomes [23]. Oxidative stress plays a crucial role in key pathophysiological processes during pregnancy, with its dysregulation contributing to hypertensive disorders and CKD.

Oxidative stress also plays a decisive role in shaping kidney development, eventually leading to kidney programming [24,25]. These morphological and functional changes can persist, increasing the risk of hypertension and adverse kidney outcomes in both mother and offspring. Given its impact, preclinical research suggests that antioxidant therapies during pregnancy and lactation may work as promising reprogramming strategies to avert kidney disease and hypertension in offspring [8].

This narrative review aims to summarize current knowledge on developmental programming mechanisms influencing maternal and offspring kidney health and hypertension, with a focus on the role of oxidative stress in preclinical models. Additionally, it explores the potential of antioxidant therapy as a reprogramming strategy for kidney disease and hypertension with developmental origins, while highlighting gaps that require further translational research.

The search was concluded in January 2025. We conducted searches in the Medline, PubMed, and Embase databases using the following keywords: “kidney disease”, “hypertension”, “nephrogenesis”, “blood pressure”, “life course”, “developmental programming”, “DOHaD”, “free radicals”, “offspring”, “progeny”, “mother”, “prenatal”, “nitric oxide”, “oxidative stress”, “pregnancy”, “lactation”, “breastfeeding”, “reprogramming”, “reactive oxygen species”, “reactive nitrogen species”, and “antioxidant”. The search was limited to English-language papers, and additional studies were identified from references of eligible articles.

## 2. Maternal–Fetal Interface and Oxidative Stress

### 2.1. Oxidative Stress in Pregnancy

During gestation, balancing ROS/RNS and antioxidants is vital for maintaining the maternal–fetal interface and promoting a healthy pregnancy [11,12]. ROS/RNS plays a role in redox signaling, but in significant concentration, they are highly reactive molecules that can cause oxidative damage. [13] Physiologically, ROS have a vital role in fetal development, contributing to processes such as oocyte development [26], blastocyst implantation [27], placental development [28], and organogenesis. ROS consist of free radicals like superoxide (O_2_^−^) and hydroxyl (OH^−^), along with non-radicals such as hydrogen peroxide (H_2_O_2_). Superoxide initiates reactions generating other ROS. RNS, including nitric oxide (NO), nitrogen dioxide (NO_2_^−^), and peroxynitrite (ONOO^−^), contribute to cytotoxicity, with peroxynitrite forming from the reaction between superoxide and NO.

Fetal oxygen needs vary by trimester, starting low in the first and increasing in the second and third due to rapid growth and placental circulation [29,30]. High oxygen consumption and metabolism elevate ROS production, but excessive ROS can disrupt these processes, leading to pregnancy complications [12]. Oxidative damage occurs when antioxidant defenses fail, with adverse conditions in pregnancy like preeclampsia [31], gestational diabetes mellitus [32], obesity [33], placental disorders [34], and intrauterine growth retardation (IUGR) [35] known to induce oxidative stress.

NO has a crucial role in regulating maternal and fetal homeostasis, essential for feto-placental blood flow and placental development [36,37]. It contributes to vascular reactivity, placental bed resistance, and angiogenesis [38,39]. However, as a free radical and weak oxidant, NO can interact with redox intermediates, causing protein oxidation, lipid peroxidation, mitochondrial dysfunction, and cell death, contributing to cytotoxic pathogenesis [40,41].

Nitric oxide synthase (NOS) catalyzes the conversion of L-arginine into NO and L-citrulline [42]. However, NOS uncoupling leads to NO depletion and increased superoxide production [43]. Asymmetric dimethylarginine (ADMA), an endogenous NOS inhibitor, can disrupt NO synthesis, but L-arginine can displace ADMA, restoring NO production. Circulating ADMA levels decrease in the first trimester but rise as pregnancy progresses [44,45]. Early in gestation, low ADMA and high NO levels facilitate hemodynamic adaptation, enhanced organ blood flow, and uterine relaxation to support fetal growth. In contrast, elevated ADMA in later gestation promotes uterine contractility, essential for labor and delivery [46]. L-arginine supplementation can compete with ADMA to enhance NO production, potentially benefiting compromised pregnancy [36,37].

In high-risk pregnancies, including gestational diabetes [47], preeclampsia [48], and gestational hypertension [49], ADMA levels are meaningfully higher than in normal pregnancy. Overall, oxidative stress—stemming from a disruption in the ROS–NO balance—plays a critical role in fetal programming in these conditions.

### 2.2. Maternal Kidney Disease and Hypertension

Pregnancy can impact kidney disease, often leading to a decline in renal function, particularly in the presence of hypertension and proteinuria. Even mild CKD significantly increases the risk of pregnancy complications, including preeclampsia, small for gestational age, and preterm birth [15].

ROS contribute to kidney disease by directly damaging kidney cells, resulting in inflammation, dysfunction, and fibrosis [50,51]. ROS also trigger signaling pathways and transcription factors that worsen these processes. Hypertension is associated with renal oxidative stress and NOS uncoupling, disrupting the ROS–NO balance [43]. This imbalance leads to endothelial dysfunction, epithelial damage, glomerular hypertension, and albuminuria [50]. Animal studies indicate that targeting ROS/RNS with antioxidants could present a potential therapeutic strategy for managing kidney disease and hypertension [8,24,51].

Hypertensive disorders of pregnancy (HDP) affect 5–10% of pregnancies and pose risks to both maternal health and fetal development [52]. HDP encompasses pre-existing hypertension, gestational hypertension, and preeclampsia/eclampsia [53]. Endothelial dysfunction, driven by oxidative stress and NO deficiency, plays an essential role in these complications [54].

Preeclampsia originates at the maternal–fetal interface, leading to hypertension, proteinuria, and potential multi-organ dysfunction, fetal growth restriction, and maternal mortality. While its pathophysiology remains poorly understood, oxidative stress is a key factor [55]. ROS may activate platelet adhesion and aggregation, resulting in intravascular coagulopathy in preeclampsia. This disrupts uteroplacental blood flow, causing placental infarction and fetal growth restriction. Antioxidant enzymes like catalase and SOD protect against oxidative damage, but placental ischemia in preeclampsia reduces their activity, increasing oxidative stress. This contributes to hypertension and proteinuria, key features of preeclampsia [56].

### 2.3. Oxidative Stress and Human Kidney Development: A Missing Link?

Human kidney development begins at weeks 3–4 of gestation and completes by around 36 weeks [57]. It encompasses three structures derived from the posterior intermediate mesoderm: the pronephros and mesonephros, which undergo regression, and the metanephros, which develops into the definitive kidneys. The ureteric bud (UB) initiates metanephric kidney development by invading the metanephric mesenchyme (MM) [58]. The MM develops into nephrons, while UB branching gives rise to the collecting ducts. Renal vesicles, derived from mesenchymal-to-epithelial transition, serve as nephron precursors. UB branching is essential for the collecting duct system and nephron formation.

The nephron is the functional unit of the kidney, with nephron endowment reflecting nephrogenesis success. Human kidneys typically have around 1 million nephrons, although this number can vary [59]. Nephrogenesis peaks between 18–32 weeks and completes by 36 weeks [60]. After birth, kidney growth continues [61]. Infants typically reach adult-like GFR between 6 and 24 months [62]. In rodents, kidney development is faster, with nephrogenesis continuing for 1–2 weeks postnatally [63]. Adverse prenatal and early postnatal conditions, such as oxidative stress, can significantly impact kidney development.

At full-term birth, neonates usually have a complete set of nephrons. However, preterm birth, IUGR, inadequate postnatal nutrition, and certain medications (such as gentamicin) may be associated with a reduced number of nephrons [20]. This deficiency contributes to glomerular hypertension, hyper-perfusion injury, and progressive nephron loss, increasing the risk of CKD [64].

Nevertheless, nephron numbers cannot be measured in vivo [60]. While prior autopsy studies estimate about 1 million nephrons per kidney, their exact count remains uncertain. Currently, human studies still lack clarity on perinatal oxidative stress-induced kidney disease and its molecular mechanisms [8,10]. Kidney biopsies in neonates are challenging, and the relationship between oxidative stress biomarkers and kidney pathologies remains unclear. As a result, much of our knowledge on renal programming and prevention comes from animal studies.

## 3. Animal Evidence for Oxidative Stress-Related Kidney Programming

Recent developments in animal models have greatly improved our understanding of the molecular mechanisms driving kidney programming. Beyond oxidative stress, key mechanisms include the abnormal activation of the renin-angiotensin system (RAS), dysregulated nutrient-sensing signals, reduced nephron number, and gut microbiota dysbiosis [6,7,8,18,19,20,25]. The strong interconnections between oxidative stress and these other core mechanisms highlight the prominent role of oxidative stress in kidney development [10].

Table 1 summarizes preclinical models of oxidative-stress-related kidney programming [65,66,67,68,69,70,71,72,73,74,75,76,77,78,79,80,81,82,83,84,85,86,87,88,89]. This review primarily focuses on adverse environmental factors that begin during gestation and lactation. A broad array of environmental stimuli can contribute to oxidative-stress-related kidney programming, including maternal nutritional imbalances [65,66,67,68,69,70,71,72,73,74,75], maternal diseases and pregnancy complications [76,77,78,79,80,81,82,83,84,85,86], medication use [87,88,89], and exposure to environmental toxins [90,91,92,93].

**Figure 1:**
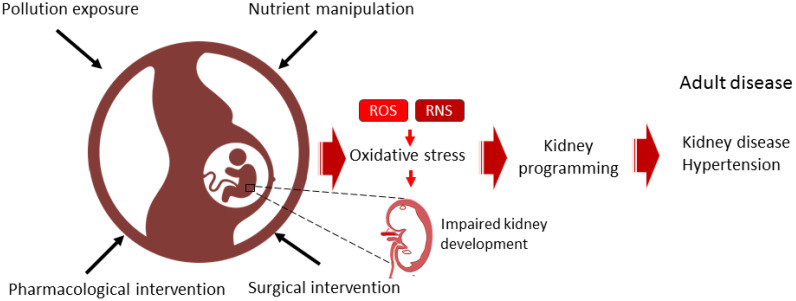
Diagram illustrating the use of dietary, surgical, pharmacological, or pollution-related interventions during pregnancy to induce oxidative stress and kidney programming, thereby increasing the risk of developing kidney disease and hypertension throughout the lifespan.

Although genetically engineered animal models can be utilized to explore the effects of prenatal oxidative stress modifications on offspring kidney outcomes [94,95], nearly all studies involving genetic alterations have focused on modifying the offspring’s genome rather than that of the parents. This makes it challenging to separate the effects of such manipulations during the prenatal period on kidney programming from the ongoing effects of genetic changes throughout the lifespan via epigenetic mechanisms. Consequently, most evidence comes from animal studies using dietary, surgical, pharmacological, or pollution interventions to induce antenatal oxidative stress programming (Table 1). Rats are the most commonly used species, followed by mice and sheep. Major adverse kidney outcomes associated with kidney programming include hypertension [67,68,69,70,71,72,73,76,77,78,79,80,81,82,83,84,85,86], tubulointerstitial injury [65,66,77,83,91], renal hypertrophy [65,66,74,81,82], reduced nephron number [65,66,77,90], albuminuria [74,75,90] kidney dysfunction [73], and glomerulosclerosis [75]. The various models employing dietary, surgical, pharmacological, or pollution-related interventions to induce oxidative stress and kidney programming, along with their interconnections with kidney disease and hypertension in later life, are depicted in Figure 1.

### 3.1. Maternal Insults

Table 1 illustrates that nutritional imbalance is the most common factor inducing kidney programming. Nutritional manipulations can be categorized into various models, including those that manipulate maternal diets through reduced calorie intake [65,66], reduced protein intake [67], excessive fructose intake [68], altered methyl donor levels [69], restricted iron intake [70], and increased fat intake [71,72,73,74,75]. Additionally, maternal diseases and pregnancy complications, such as reduced uterine perfusion [76], diabetes [77,78], preeclampsia [79,80], chronic kidney disease (CKD) [81,82], endothelial dysfunction [83,84], inflammation [85,86], and maternal stress [87,88,89], can be induced by surgical or pharmacological interventions. Various other antenatal pollution exposures, such as nicotine [90], di-n-butyl phthalate [91], bisphenol A (BPA) [92], and 2,3,7,8-tetrachlorodibenzo-p-dioxin (TCDD) [93], can also lead to kidney programming through oxidative stress.

Antenatal programmed oxidative stress modifications can also make both the mother and offspring more susceptible to subsequent programming insults that directly contribute to hypertension and CKD risk factors, often termed “second hits”. It is possible that many prenatal programming factors need further negative exposures throughout life to fully reveal kidney programming effects. This concept is supported by 2-hit models shown in Table 1, such as a rat model of antenatal BPA exposure followed by a high-fat diet [92] and antenatal TCDD exposure followed by dexamethasone exposure [93]. As a result, the heightened sensitivity of both the mother and offspring to future stressors represents a critical, yet frequently underestimated, consequence of prenatal oxidative stress manipulation.

### 3.2. Oxidative Stress Programming Mechanisms

Kidney programming can be linked to various oxidative stress-driven mechanisms, including increased ROS [76,83,89,90,91], decreased antioxidant capabilities [67,73,88], reduced NO bioavailability [65,66,68,71,77,80,81,82,84,87,89,92,93], and increased oxidative damage [65,66,67,68,69,70,71,72,73,74,75,76,77,78,79,81,82,85,86,87,88,92,93].

Since assessing ROS in human kidneys is challenging, animal studies have provided substantial evidence linking elevated renal ROS levels to adverse kidney outcomes in models of reduced uterine perfusion [76], maternal angiotensin II administration [83], antenatal glucocorticoid administration [89], maternal nicotine exposure [90], and prenatal di-n-butyl phthalate exposure [91]. Conversely, impaired antioxidant defenses have also been implicated in kidney programming in models of low protein intake [67], high-fat diet [73], and antenatal glucocorticoid administration [87].

When an accumulation of ROS or RNS under detrimental intrauterine conditions exceeds antioxidant defenses, oxidative damage occurs, impairing kidney development. Oxidative damage is typically assessed by measuring lipid peroxidation products such as malondialdehyde (MDA), F2-isoprostanes (F2-IsoPs), and thiobarbituric acid-reactive substances (TBARSs) [96]. Elevated MDA levels have been observed in models of high-fat diet [72], LPS administration [86], and antenatal glucocorticoid administration [87]. Additionally, increased F2-IsoP levels have been linked to kidney programming in adult offspring in models of low-protein diet [67] and reduced uterine perfusion [76]. In a rat model of maternal diabetes induced by streptozotocin, elevated TBARSs have been associated with kidney oxidative damage and offspring hypertension [78].

In addition, oxidative DNA damage can be assessed by measuring 8-hydroxy-2′-deoxyguanosine (8-OHdG), an oxidized nucleoside released during the repair of damaged DNA [97]. Elevated 8-OHdG expression in rat offspring kidneys has been associated with adverse kidney outcomes in various models, including caloric restriction [65,66], protein restriction [67], high-fructose diet [68], high-methyl-donor diet [69], methyl-deficient diet [69], iron deficiency diet [70], high-fat diet [73], maternal CKD [81,82], antenatal glucocorticoid administration [88], prenatal BPA exposure combined with a high-fat diet [92], and prenatal TCDD plus dexamethasone exposure [93].

Furthermore, several studies in Table 1 suggest that disruption of the ADMA–NO balance plays a role in oxidative stress-induced kidney programming [65,66,68,71,77,80,81,84,87,89,92]. Mounting evidence highlights the key role of epigenetic regulation in fetal programming [98]. ROS and NO influence key epigenetic processes, including DNA methylation, histone modifications, and microRNA regulation [99,100]. ADMA reduces NO production and increases ROS [42], leading to a dose-dependent reduction in nephron numbers in embryonic kidneys [101]. Transcriptome analysis of embryonic kidneys exposed to 10 µM ADMA identified 1221 differentially expressed genes (DEGs; 735 upregulated, 486 downregulated) [101], many linked to kidney development and epigenetic regulation. Similarly, maternal NO inhibition with L-NAME resulted in 2289 DEGs (1259 upregulated, 1030 downregulated) in neonatal kidneys [79]. These findings propose that oxidative stress during gestation contributes to kidney programming and increases the risk of kidney disease in offspring.

As shown in Table 1, different maternal insults can lead to similar adverse kidney outcomes in adult offspring, suggesting common mechanisms—such as oxidative stress—may underlie kidney programming. A deeper understanding of oxidative stress-induced kidney programming could help identify modifiable risk factors during pregnancy and facilitate the development of targeted interventions to prevent and treat kidney disease.

## 4. Antioxidants as a Strategy for Prevention and Treatment

As mentioned, perinatal oxidative stress plays a key role in kidney programming, increasing the risk of adult kidney disease. While antioxidant therapies may counteract excess ROS or RNS, their clinical benefits in kidney disease and hypertension remain inconclusive [102,103,104]. However, preclinical models in DOHaD-related disorders suggest potential benefits of early-life antioxidant therapy, which can be obtained through diet or synthetic sources.

For a substance to qualify as a dietary antioxidant, it must be commonly found in human diets and proven to reduce ROS and RNS in humans—not just in vitro. The Food and Nutrition Board of the U.S. National Institute of Medicine defines dietary antioxidants as food components that significantly reduce oxidative damage based on three criteria: presence in the human diet, measurable levels in common foods, and proven physiological effects [105].

**Figure 2:**
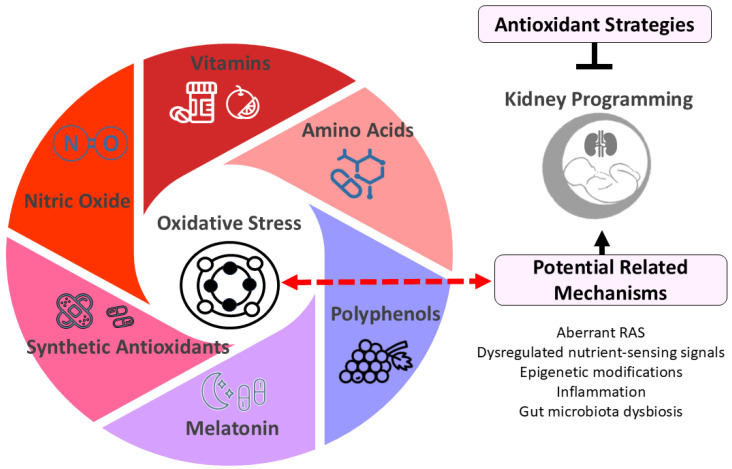
Diagram illustrating potential antioxidant strategies for kidney programming. Oxidative stress plays a crucial role in kidney programming, potentially interacting with other factors such as dysregulated renin-angiotensin system (RAS) and nutrient-sensing signals to impact kidney development. Early-life antioxidant interventions have shown promise in preventing and treating kidney programming-related disorders. These antioxidant strategies include vitamins (e.g., vitamins C and E), amino acids (e.g., L-arginine and L-citrulline), polyphenols (e.g., resveratrol), melatonin, synthetic antioxidants (e.g., N-acetylcysteine), and therapies targeting nitric oxide pathways.

Dietary antioxidants can be either water- or lipid-soluble. Lipid-soluble antioxidants protect cell membranes, while water-soluble ones act in the cytosol, mitochondria, or extracellular fluids. Common water-soluble antioxidants include vitamin C, glutathione, uric acid, and lipoic acid, while lipid-soluble antioxidants include vitamins A and E, coenzyme Q, carotenoids, and polyphenols. Synthetic antioxidants are man-made compounds designed to neutralize free radicals and reduce oxidative damage. Examples include N-acetylcysteine (NAC) and MitoQ. Additionally, certain amino acids (e.g., L-arginine and L-citrulline) and hormones (e.g., melatonin) have been used in animal models of kidney programming due to their antioxidant properties. The following section briefly introduces antioxidants studied in kidney programming (Figure 2).

### 4.1. Vitamins

The antioxidant properties of various vitamins may provide potential benefits in CKD. These vitamins include vitamins A, C, and E, along with selenium and folic acid [106]. Among them, vitamins C and E are the most commonly used. Vitamin C neutralizes free radicals as a water-soluble antioxidant [107], while vitamin E, a lipid-soluble antioxidant, reduces ROS production by inhibiting oxidative enzymes [108].

In a rat model of maternal caloric restriction, offspring hypertension was prevented by the combined supplementation of vitamins C and E, selenium, and folic acid [109]. Additionally, gestational supplementation with vitamin C or E protected against hypertension in offspring exposed to maternal LPS administration [110,111].

However, a meta-analysis has linked high doses of vitamin A, β-carotene, and vitamin E to increased mortality [112], while excessive vitamin A intake has been related to birth defects [113]. Therefore, perinatal vitamin supplements should only be used in cases of deficiency, not routinely. Furthermore, contamination of vitamin supplements, particularly with heavy metals and toxins, poses a concern, especially for the vulnerable fetus. Ensuring these supplements are free from such contaminants is crucial for maternal and fetal health [114].

### 4.2. Polyphenols

Polyphenols are widely recognized dietary antioxidants. These naturally occurring plant compounds exhibit antioxidant properties and function as metal chelators, free radical scavengers, NOS activators, and stimulators of antioxidant enzymes [115]. Polyphenols have been studied for their potential role in improving kidney health [116,117]. While maternal polyphenol supplementation has shown potential benefits for pregnancy and fetal outcomes [118,119], evidence from human studies on its long-term effects on offspring kidney health remains limited [120,121].

Polyphenols are classified into flavonoids and nonflavonoids [115]. Quercetin, a flavonoid antioxidant, protected adult rat progeny from high-fat maternal-diet-induced kidney programming and hypertension [122]. Similarly, epigallocatechin gallate, used during gestation and lactation, moderated hypertension in a rat model of prenatal dexamethasone exposure [123].

Resveratrol is among the most extensively studied nonflavonoid polyphenols due to its broad spectrum of potential health benefits [124]. Its antioxidant properties include scavenging ROS and RNS, enhancing antioxidant enzyme activity, and increasing glutathione levels [125]. In rat models of kidney programming—such as high-fructose diet [71], maternal CKD [82], maternal ADMA administration [84], prenatal BPA exposure combined with a high-fat diet [92], and prenatal TCDD plus dexamethasone exposure [93]—resveratrol has demonstrated protective effects on kidney health in adult offspring.

For example, perinatal resveratrol therapy protected offspring from kidney programming induced by maternal CKD through reducing renal 8-OHdG expression and increasing NO production [66]. However, a key limitation of polyphenols in clinical applications is their low bioavailability [126]. Given the interindividual variability and the complexity of polyphenol pharmacokinetics, further research is crucial to clarify their effects on kidney health, particularly in pregnant women and their children.

The Mediterranean diet, one of the most widely studied dietary patterns with antioxidant properties, has been shown to be beneficial for cardiovascular and kidney health [127,128,129]. These effects are largely attributed to bioactive ingredients such as polyphenols [130]. The Mediterranean diet is characterized by high intakes of grains, vegetables, fruits, fish, legumes, and olive oil [127]. For example, polyphenol-rich olive oil has been found to counteract high-fat diet-induced oxidative stress, thereby preventing the progression of kidney disease and hypertension in spontaneously hypertensive rats (SHRs) [131]. Thus, specific diets (e.g., the Mediterranean diet) or foods (e.g., olive oil) containing polyphenols may hold promise for the prevention and treatment of kidney programming-related disorders.

### 4.3. Amino Acids

Several amino acids exhibit antioxidant properties [132] and have demonstrated therapeutic and protective effects in kidney diseases [133]. L-arginine, a substrate for NOS, plays a crucial role in NO production, while L-citrulline serves as its precursor [105,106]. Given the impact of NO deficiency on kidney programming, the perinatal use of these amino acids has been explored for their potential to protect offspring against kidney disease later in life [134,135].

In human kidneys, L-citrulline is converted to L-arginine [135], and oral supplementation of L-citrulline bypasses hepatic metabolism, effectively increasing L-arginine and NO levels. Supplementation with L-citrulline during gestation and lactation has been shown to enhance NO bioavailability and protect adult offspring from kidney programming in oxidative stress-related models, including maternal caloric restriction [65], streptozotocin-induced diabetes [77], and antenatal dexamethasone exposure [88].

Moreover, L-tryptophan and L-cysteine have been evaluated as reprogramming interventions to mitigate oxidative stress and offspring hypertension in maternal CKD-induced kidney programming models [136,137]. Similarly, perinatal L-taurine supplementation prevented maternal diabetes-induced offspring hypertension by reducing oxidative stress [138]. Other amino acids, such as branched-chain amino acids (BCAAs), show potential benefits for kidney health [134], but their specific role in reducing oxidative stress requires further investigation.

### 4.4. Melatonin

Melatonin is not typically classified as a dietary antioxidant, though it has strong antioxidant properties [139]. While small amounts can be obtained from food sources like eggs, fish, nuts, and some herbs [140], its primary source in humans is endogenous synthesis by the pineal gland, making it more accurately described as a biological antioxidant rather than a dietary one.

As a potent antioxidant derived from tryptophan [141], melatonin plays a crucial role in pregnancy and fetal development [142]. It neutralizes ROS and RNS, enhances antioxidant enzyme activity, and improves NO bioavailability [143,144]. Due to these properties, melatonin and its metabolites have been explored as antioxidant therapy for pregnant women and neonates [145,146].

Some human studies have shown that melatonin treatment helps reduce oxidative stress in newborns experiencing conditions such as sepsis, asphyxia, or other disorders associated with excessive ROS production [146]. Additionally, the urinary excretion of melatonin’s metabolite could act as a biomarker for infants with IUGR, indicating its involvement in fetal programming [147].

Animal studies indicate that perinatal melatonin therapy may prevent adult-onset diseases, including kidney disease [148]. Melatonin administration in mother dams has shown renal benefits in oxidative stress-related kidney programming models, including low caloric diet [66], methyl donor diet [69], maternal L-NAME administration [79], and high-fructose diet [149]. Its protective effects include reduced ADMA [66], lower 8-OHdG expression [69], decreased lipid peroxidation [79], and enhanced NO levels [149].

While melatonin is generally considered safe for children [145,146], its use during pregnancy remains unadvised due to a lack of sufficient evidence supporting its safety [150]. While animal studies suggest melatonin may protect kidney health by mitigating oxidative stress-related diseases, further research is required to confirm its safety, efficacy, and role as an epigenetic regulator before clinical recommendations can be made [151,152].

### 4.5. Synthetic Antioxidants

In addition to natural dietary antioxidants, several synthetic antioxidants have been studied in preclinical models of kidney programming. NAC is a widely studied synthetic antioxidant [153]. It serves as a precursor to glutathione and an L-cysteine analogue for hydrogen sulfide (H_2_S) synthesis [154]. NAC has demonstrated therapeutic potential in neonatal kidney disease in rat sepsis [155] and porcine neonatal asphyxia models [156], though human studies remain limited. NAC treatment during gestation and lactation protected rat offspring from maternal L-NAME-induced kidney programming by enhancing the expression and activity of renal H_2_S-generating enzymes [79]. In an antenatal dexamethasone and postnatal high-fat diet model [157], NAC’s protective effects were linked to increased plasma glutathione levels and upregulation of H_2_S-producing enzymes. Additionally, NAC prevented maternal suramin-induced offspring hypertension by boosting glutathione production, restoring NO levels, and activating the H_2_S pathway [80].

Other synthetic antioxidants, such as MitoQ and dimethyl fumarate (DMF), have also been explored in kidney disease models [158,159]. MitoQ, a coenzyme Q10 analogue, reduces oxidative stress by inhibiting superoxide production and lipid peroxidation [160]. Perinatal MitoQ treatment prevented hypertension, nephron loss, and kidney injury in a maternal nicotine exposure model [90]. DMF, an Nrf2 activator, reduced oxidative stress in a prenatal dexamethasone and postnatal high-fat diet model by lowering ADMA and 8-OHdG levels while increasing NO [161].

Additionally, SOD mimetics like tempol, when used during gestation, reduced proteinuria and BP in adult SHR offspring [162]. However, none of these synthetic antioxidants have been adopted in clinical practice, highlighting the need for further research.

### 4.6. Nitric Oxide-Targeted Therapies

Impaired NO action is linked to increased oxidative stress, making the restoration of NO bioavailability a key strategy for enhancing antioxidant defenses [163]. NO levels can be improved through NOS substrate supplementation, ADMA inhibition, NO donor or nitrodilator administration, and NOS enhancement [164].

As discussed earlier, supplementing L-arginine and L-citrulline can enhance NO production. Additionally, ADMA-lowering agents can help restore ROS/NO balance, although no specific drug is currently available [165,166]. Several clinically used drugs lower ADMA levels, including telmisartan [167], glucagon-like peptide-1 receptor agonists [168], rosuvastatin [169], and epigallocatechin-3-gallate [170], which reduce ADMA-generating enzyme expression. Others, such as melatonin [66], NAC [80], metformin [171], atorvastatin [172], salvianolic acid A [173], telmisartan [167], oxymatrine [174], rosuvastatin [169], Farnesoid X receptor agonists [175], and nebivolol [176], enhance ADMA metabolism. Among them, only NAC and melatonin have been studied in kidney programming models to prevent offspring hypertension. Hence, further research is needed to explore their potential antioxidant benefits for kidney health.

Despite advancements in NO donors [177], their role in kidney programming remains largely unexplored. Similarly, few nitrodilators have been investigated in this context. Nitrodilators, such as pentaerythritol tetranitrate (PETN), nitroglycerin, and molsidomine, mimic NO’s vasodilatory effects by releasing NO from external sources [178,179]. PETN and molsidomine have shown benefits against hypertension in SHRs and fawn-hooded hypertensive rats, respectively [180,181].

While NO-related reprogramming interventions show promise in improving kidney health in preclinical studies, their interaction with oxidative stress mechanisms in kidney programming remains unclear. Further research is required to determine optimal dosage and duration before clinical translation.

### 4.7. Others

Evidence supports the beneficial effects of short-chain fatty acids (SCFAs) in modulating oxidative stress, which plays a role in CKD pathogenesis and progression [182]. SCFAs, including acetate, propionate, and butyrate, are metabolites produced by gut bacteria during the fermentation of dietary fibers [183].

While SCFAs are not classified as traditional antioxidants, they possess indirect antioxidant properties. For instance, butyrate inhibits histone deacetylases (HDACs), promoting anti-inflammatory pathways and enhancing the expression of antioxidant enzymes such as SOD and glutathione peroxidase [184]. While SCFAs do not directly scavenge free radicals like conventional antioxidants, they help lower oxidative stress and support cellular health. Since SCFAs influence fetal development and programming in pregnancy [185], animal studies suggest that perinatal SCFA supplementation may prevent hypertension and kidney disease in adult offspring across various kidney programming models [186]. However, whether their protective effects stem primarily from antioxidant actions remains to be determined.

## 5. Research Gaps and Future Directions

Manipulating redox status during specific stages of gestation to influence offspring kidney development is challenging due to biological and technical complexities. Pregnancy involves dynamic redox changes, and disruptions can lead to multi-organ dysfunction in the developing fetus, impaired placental function, and pregnancy complications. Severe outcomes, including failed kidney development, may result, posing significant risks to offspring survival.

Animal studies suggest that oxidative stress—whether induced by dietary, surgical, pharmacological, or pollution interventions—contributes to kidney programming and increases the risk of kidney disease and hypertension. Since vulnerability to oxidative stress varies across organs, different maternal insults may lead to distinct programming effects. Key questions remain: (1) Does oxidative stress alone drive kidney programming, and when do changes occur? (2) Which free radical signals trigger lifelong redox alterations linked to kidney disease? (3) Are these changes organ-specific and reversible?

Although definitive answers remain elusive, emerging evidence provides some insights. Oxidative stress is a key contributor to kidney programming but not its sole determinant. Other factors, such as dysregulated nutrient-sensing signals [19], epigenetic modifications [98], inflammation [106], gut microbiota dysbiosis [186], and aberrant RAS [187], may interact with oxidative stress to influence kidney development. The timing of these changes likely occurs during critical windows of gestation, particularly in mid-to-late pregnancy when nephrogenesis is most active. However, oxidative stress in early pregnancy and the postnatal stage may also modify redox status and kidney programming.

Several key factors, such as superoxide and ADMA, can disrupt redox balance and alter gene expression related to nephron formation [10], though their relative importance requires further investigation. Some redox-induced changes may be reversible through postnatal interventions, such as antioxidant supplementation [8]. However, structural alterations in the kidney may have long-term consequences that are more difficult to reverse once nephrogenesis is complete [25]. Further research is needed to define critical developmental windows and redox-sensitive pathways that may serve as therapeutic targets.

Although rodents are widely used in kidney programming studies [188], their placentation and fetal development differ significantly from humans [189]. While non-human primates are the gold standard due to their genetic and biological similarity to humans, rodents remain the most commonly used models in DOHaD research [188] due to their low cost, short life cycle, and ease of genetic modification. Other species, such as rabbits, sheep, pigs, and cows, offer advantages depending on the study focus. Rabbits share similarities in lipid metabolism and placental structure with humans, pigs are ideal for early fertilization studies, sheep have a long gestation and fetal size comparable to humans, and cows, as large monotocous animals, also have a prolonged gestation period [190].

Non-rodent model organisms like zebrafish, Drosophila, and C. elegans provide valuable insights into oxidative stress research [191], offering advantages such as high reproduction rates, small size, live imaging capabilities, and ease of gene manipulation. However, their application in DOHaD research remains limited. When selecting an animal model, factors such as genetic background, anatomy, physiology, gestation length, litter size, life cycle, and relevance to study mechanisms must be considered. Findings from rodent studies should be interpreted with caution, and large animal models, which better mimic human physiology, should not be overlooked.

Another key research gap is the methodological limitations in assessing oxidative stress. Accurate quantification of redox components requires well-validated methods to ensure rigor, reproducibility, and comparability across studies. Analyzing isolated markers may misrepresent the functional status of the redox system, highlighting the need for comprehensive pathway analysis.

Currently, oxidative stress in kidney programming is assessed through biomarkers of ROS, NO, RNS, oxidation by-products, and antioxidants [192]. However, no universally accepted biomarker panel exists, leading to variability in study results. Many biomarkers (e.g., MDA, F2-isoprostanes, 8-OHdG) measure oxidative damage rather than oxidative stress itself. Additionally, the short half-lives of ROS/RNS make direct measurement difficult, forcing reliance on indirect markers that may not fully capture oxidative stress dynamics [193].

While preclinical studies support antioxidant strategies for kidney health, clinical validation is needed. Determining the optimal antioxidant and therapeutic dose remains crucial to translating animal research into human benefits. Extensive cohort studies involving pregnant women are necessary to determine the causal relationship between perinatal antioxidant supplementation and kidney outcomes in offspring. So far, there have been more than 200 trials studying the effects of antioxidants on pregnant women [194]. However, none of them focused on offspring kidney health. Given the challenges of recruiting pregnant women and neonates for research, breastmilk could serve as a starting point. Breastmilk has a strong antioxidant profile [195,196], and since the WHO recommends exclusive breastfeeding for the first six months [197], its potential role in protecting against renal programming warrants further investigation.

The safety of antioxidant supplements is another concern, as some may act as pro-oxidants under certain conditions [198]. For instance, vitamin E can become a radical if insufficient vitamin C is available for its regeneration [199]. The pro-oxidant effects of antioxidants depend on their concentration, highlighting the need for supplementation only when oxidative stress is confirmed. While oxidative damage can be assessed in animal models, human studies—especially in fetuses and neonates—are limited. Antioxidants may also affect healthy tissues, underscoring the importance of maintaining a balanced ROS/RNS state. Despite advances in oxidative stress biomarkers, their role in predicting adult-onset kidney diseases remains unclear [200]. These key research gaps are summarized in Table 2.

## 6. Conclusions

Despite the high prevalence of hypertension and kidney disease, as well as increased perinatal mortality, global screening practices for pregnant women remain underutilized [201,202,203]. In humans, there is limited understanding of how early-life environmental factors influence oxidative stress and kidney programming, which may increase the risk of hypertension and CKD later in life. Although animal studies have provided valuable insights into adverse kidney outcomes in both dams and offspring, and antioxidant strategies show promise, further research is needed to translate these findings into clinical practice.

## Figures and Tables

**Table 1:** Overview of Animal Models of Kidney Programming Related to Oxidative Stress.

Animal Models	Species	Programming Exposure	Mechanisms of Oxidative Stress	Ref.
Nutrient manipulation	Rat	Caloric restriction	↓ NO, ↑ Kidney oxidative damage	[65,66]
	Rat	Protein restriction	↑ Oxidative damage, ↓ Antioxidant capabilities	[67]
	Rat	High-fructose diet	↓ NO, ↑ Kidney oxidative damage	[68]
	Rat	Methyl-deficient diet	↑ Kidney oxidative damage	[69]
	Rat	High-methyl-donor diet	↑ Kidney oxidative damage	[69]
	Rat	Low-iron diet	↑ Kidney oxidative damage	[70]
	Rat	High-fat diet	↓ NO, ↑ Kidney oxidative damage, ↓ Antioxidant capabilities	[71,72,73]
	Mouse	High-fat diet	↑ Kidney oxidative damage, ↑ ROS-generating enzymes	[74,75]
Surgical intervention	Rat	Reduced uterine perfusion	↑ Kidney oxidative damage, ↑ ROS	[76]
Pharmacological intervention	Rat	Diabetes induced by streptozotocin	↓ NO, ↑ Kidney oxidative damage	[77,78]
	Rat	Preeclampsia induced by L-NAME	↑ Kidney oxidative damage	[79]
	Rat	Preeclampsia induced by suramin	↓ NO	[80]
	Rat	CKD induced by adenine	↓ NO, ↑ Kidney oxidative damage	[81,82]
	Rat	Endothelial dysfunction induced by angiotensin II	↑ ROS	[83]
	Rat	Endothelial dysfunction induced by ADMA	↓ NO	[84]
	Rat	LPS administration	↑ Kidney oxidative damage	[85,86]
	Rat	Antenatal glucocorticoids	↓ NO, ↑ Kidney oxidative damage, ↓ Antioxidant capabilities	[87,88]
	Sheep	Antenatal glucocorticoids	↓ NO, ↑ ROS	[89]
Pollution exposure	Mouse	Maternal nicotine exposure	↑ Kidney ROS	[90]
	Rat	Maternal di-n-butyl phthalate exposure	↑ Kidney ROS	[91]
	Rat	Maternal bisphenol A exposure plus high-fat diet	↓ NO, ↑Kidney oxidative damage	[92]
	Rat	Prenatal TCDD plus dexamethasone exposure	↑Kidney oxidative damage, ↓ NO	[93]

↑ = increased; ↓ = decreased.

**Table 2:** Summary of Key Research Gaps.

Key Research Gap	Summary
Challenges in manipulating redox status during gestation	Redox changes during pregnancy are dynamic.
	Disruptions can cause multi-organ dysfunction, failed kidney development, and even fetal death.
Organ-specific vulnerability	Uncertainty about whether oxidative stress alone drives kidney programming. Uncertainty about when critical changes occur.
	Uncertainty about whether effects are organ-specific and reversible.
Limitations of rodent models	While rodents are commonly used, their placental and fetal development differ from humans.
	Large animal models, which better mimic human physiology, should be considered.
Methodological challenges in assessing oxidative stress	Current biomarkers primarily measure oxidative damage rather than oxidative stress itself.
	Short-lived ROS/RNS make direct measurement challenging
Antioxidant interventions	Unclear optimal antioxidant type, dosage, and timing for kidney protection.
	Limited human studies to confirm benefits seen in preclinical research.
Safety concerns with antioxidants	Some antioxidants may act as pro-oxidants under certain conditions.
	Need for careful evaluation of risks, especially in fetuses and neonates.
Long-term impact on kidney health	Lack of data on whether oxidative stress biomarkers predict adult-onset kidney diseases.
	Lack of data on how early interventions influence long-term outcomes.

## Data Availability

Data are contained within the article.
